# ENT and mucopolysaccharidoses

**DOI:** 10.1186/s13052-018-0555-0

**Published:** 2018-11-16

**Authors:** Pier Marco Bianchi, Renato Gaini, Silvano Vitale

**Affiliations:** 1 Surgery Department, Otorhinolaryngology Unit, Bambino Gesù Paediatric Hospital, Scientific Research Institute, P.zza S.Onofrio, 4-00165 Rome, Italy; 2 ENT Department, S. Gerardo Hospital, Monza, Italy; 3 ENT Department, V. Fazzi Hospital, Lecce, Italy

**Keywords:** Mucopolysaccharidoses, Airway obstruction, Obstructive sleep apnoea, Otitis media with effusion, Hearing loss, Tracheotomy, Adenotonsillectomy

## Abstract

The mucopolysaccharidoses (MPS) are a heterogeneous group of inherited metabolic disorders, each associated with a deficiency in one of the enzymes involved in glycosaminoglycan (GAG) catabolism. Over time, GAGs accumulate in cells and tissues causing progressive damage, a variety of multi-organ clinical manifestations, and premature death. Ear, nose, and throat (ENT) disorders affect more than 90% of MPS patients and appear in the early stage of MPS; also reported are recurrent otitis media and persistent otitis media with effusion, macroglossia, adenotonsillar hypertrophy, nasal obstruction, obstructive sleep apnoea syndrome (OSAS), hearing loss, and progressive respiratory disorders. Undiagnosed MPS patients are frequently referred to otolaryngologists before the diagnosis of MPS is confirmed. Otolaryngologists thus have an early opportunity to recognize MPS and they can play an increasingly integral role in the multidisciplinary approach to the diagnosis and management of many children with MPS. The ENT commitment is therefore to suspect MPS when non-specific ENT pathologies are associated with repeated surgical treatments, unexplainable worsening of diseases despite correct treatment, and with signs, symptoms, and pathological conditions such as hepatomegaly, inguinal hernia, macrocephaly, macroglossia, coarse facial features, hydrocephalous, joint stiffness, bone deformities, valvular cardiomyopathy, carpal tunnel syndrome, and posture and visual disorders.

## Background

### Why is the paediatric otolaryngologist involved and what is their role in mucopolysaccharidosis?

Ear, nose, and throat (ENT) manifestations in mucopolysaccharidosis (MPS) are due to the accumulation of glycosaminoglycans (GAGs) in the head and neck region. The ENT manifestations are very common and include protruding or depressed frontal bone, a depressed nasal bridge, wide nasal alae, thick lips, angled and hypoplastic mandible (micrognathia), macroglossia, distorted teeth, gingival hypertrophy, and also restriction of the mouth opening, adenotonsillary hypertrophy, and thickening of soft tissues in the laryngopharynx. Head and neck disorders affect more than 90% of MPS patients [1, 2]. The median age of a first ENT visit is 4.2 years, generally prior to MPS diagnosis [3–9]. Symptoms such as “noisy breathing”, sleep apnoea, frequent respiratory and ear infections, chronic nasal discharge, and enlargement of the tongue, tonsils, and adenoids may often predate a definitive MPS diagnosis by several years, particularly in patients with an attenuated phenotype. MPS, owing to the non-specific nature of the early symptoms, is therefore often unrecognized [10, 11]. The early recognition and prompt diagnosis of MPS disorders is crucial. Early treatment considerably improves patient outcomes during long-term therapy and is crucial to slow disease progression before irreversible damage occurs. Undiagnosed patients are frequently referred to otolaryngologists for ENT manifestations; these specialists thus have an early opportunity to recognize MPS. Therefore, the otolaryngologist is an essential member of the care management team for the disorders of MPS patients and they are in a prime position to initiate diagnostic work-up and referral for definitive testing [9].

ENT disease in MPS can be schematically divided into respiratory disorders and otological or hearing problems.

In summary, otolaryngologists often see as yet undiagnosed MPS patients. Their careful history collection and clinical evaluation may play an important role in the diagnosis and management plan.

### What are the respiratory disorders in MPS?

**Fig. 1:**
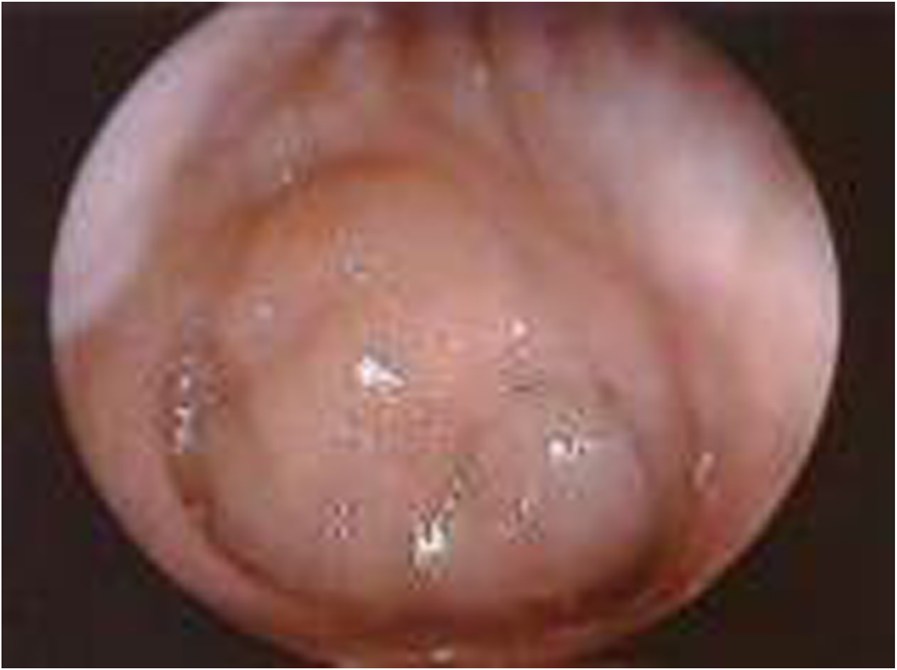
Adenoid hypertrophy

**Fig. 2:**
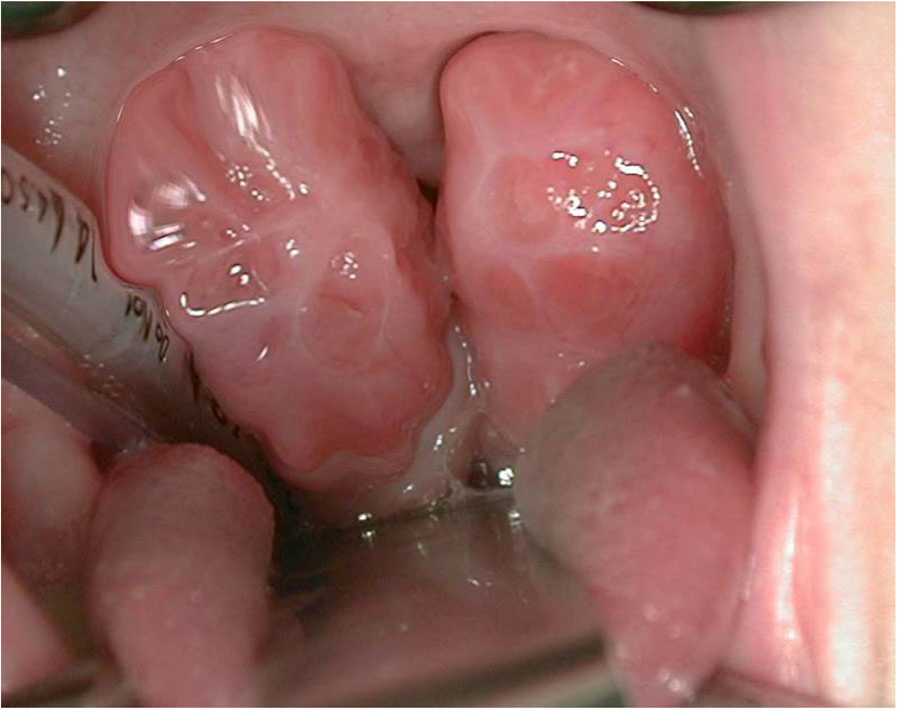
Tonsillar hypertrophy

**Fig. 3:**
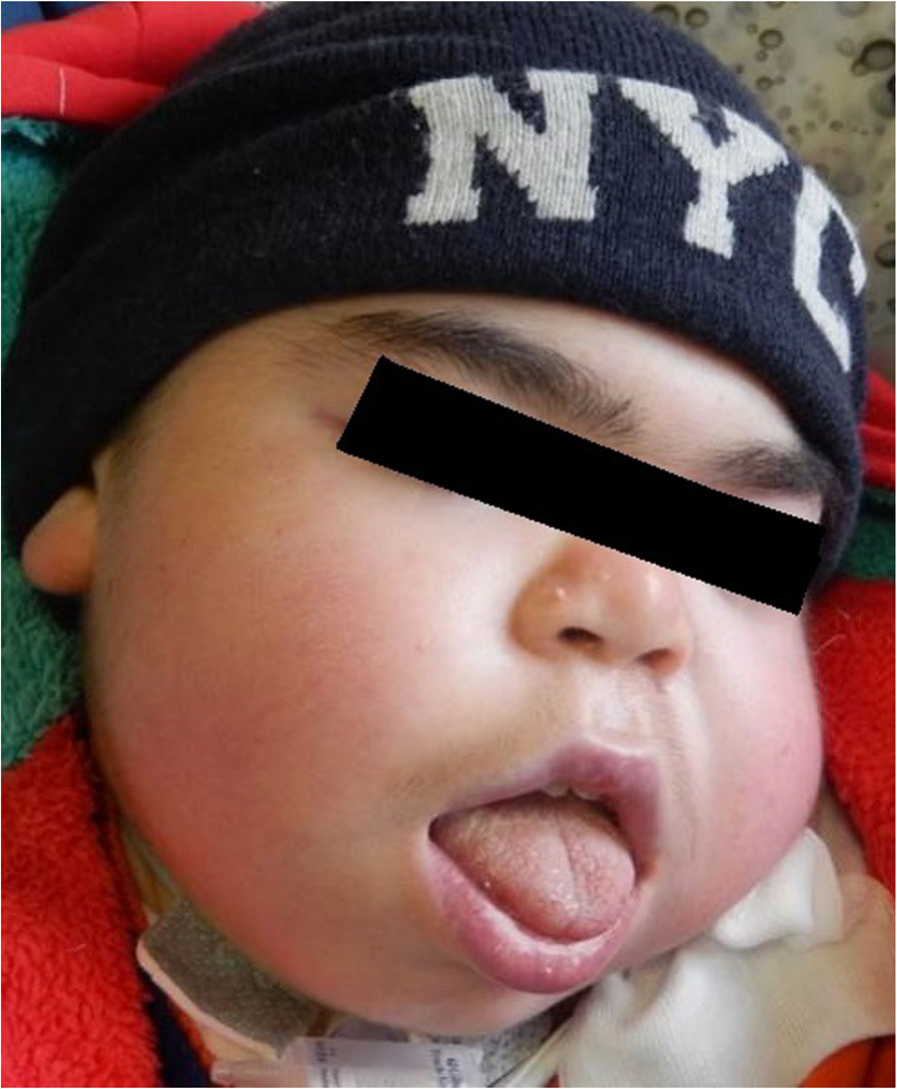
Macroglossia and typical facies

Respiratory disorders occur in all type of MPS. Airway problems result from a combination of tissue storage of GAGs that produce a distortion of airway anatomy and function [10, 12–16]. Affected individuals classically have a number of anatomical features predisposing to airway dysfunction [17]. Progressive airway damage develops, often involving multiple levels within the upper respiratory tract, secondary to the progressive deposition of GAGs within the airway [18], at any level from the “lips to lungs” [19]. Airway problems include obstructive sleep apnoea (OSA), frequent respiratory infections, adenoid (Fig. 1) and tonsillar hypertrophy (Fig. 2), irregular nasal septum, turbinate hypertrophy, macroglossia (Fig. 3), thickened pharyngeal wall, laryngeal abnormalities, tracheomalacia, tracheal stenosis and short neck, dyspnoea, restricted joint mobility, skeletal abnormalities, and increased mucus secretions in the upper and lower airways [20–24]. Chronic rhinosinusitis and chronic otitis media may occur and produce hearing impairment [10, 12]. In extreme cases, excessive tissue on the arytenoid cartilages and aryepiglottic folds can prolapse into the laryngeal inlet causing stridor and airway obstruction [24]. Furthermore, hepatosplenomegaly may limit diaphragmatic excursion, and interstitial pulmonary GAG deposits may result in a diffusion defect [13]. Thickened and copious secretions throughout the upper and lower respiratory tracts are also commonly found and there is a tendency for frequent upper and lower respiratory tract infections. Recurrent throat and ear infections are present in more than 50% of patients [25]. However, due to the low number of patients, no conclusions can be made regarding the prevalence and severity of respiratory problems for each MPS type [18]. Initially, obstructive symptoms are more pronounced in the upper airway; tracheobronchial manifestations occur later. The respiratory involvement is usually progressive and can result in morbidity and mortality early in childhood [10, 26].

The respiratory disorders in MPS can be divided into airway abnormalities (extrathoracic and intrathoracic), and alterations in respiratory and sleeping mechanics [18].

Extrathoracic anatomical abnormalities, including abnormal cervical vertebrae, a short neck, a relatively high epiglottis, deep cranial fossa narrowing the nasopharynx, a hypoplastic mandible with a short ramus, temporomandibular joint ankylosis, a small thoracic cage, frequently complicated by kyphoscoliosis [13, 27, 28], and mucopolysaccharide infiltration of the nasopharyngeal, oropharyngeal, hypopharyngeal, and laryngeal tissues, are typically seen in children with MPS and predispose them to upper airway obstruction [29]. Intrathoracic airway obstruction is also a common complication, and tracheobronchial abnormalities due to enlarged cartilage cells or soft-tissue growths can also cause narrowing of the tracheal lumen [18]. The trachea can be narrow, tortuous, or occluded by the accumulation of soft tissue [29]. Semenza et al. [17] also suggested alveolar and interstitial pulmonary involvement by GAG deposition. Depending on the site and severity of obstruction, patients may present with stridor, dyspnoea, retractions, cough, cyanosis, or difficulty with feeding [26], and the severity of respiratory dysfunction varies according to the MPS type [29].

Multiple abnormalities in MPS patients can reduce the ventilator capacity, manifesting as a reduction in vital capacity [18]. Short stature [30], kyphoscoliosis, and pectus carinatum are common, and diaphragm excursion may be compromised by hepatosplenomegaly [13, 30]. Tracheal distortion is characteristic for MPS and may reflect a disproportionate length of the trachea relative to the shortened spinal length [31, 32]. In combination with laxity of the tracheal tissue, it can cause airway collapse [31]. Cervical or lumbar spinal cord lesions may weaken expiratory muscles, impair cough, and reduce secretion clearance, predisposing patients to recurrent pneumonia. Diaphragmatic weakness may result from spinal cord compression above the phrenic nerve origin [18]. The combined effect of these alterations in respiratory mechanics together with airway abnormalities may lead to respiratory failure. Airway obstruction and parenchymal abnormality can impair pulmonary O_2_ uptake and CO_2_ excretion [18]. Ventilatory dysfunction is exacerbated by normal sleep mechanisms that increase upper airway collapsibility [33]. The severity of respiratory damage varies according to the MPS type [3, 9, 17, 29, 34–38] (Table 1). The prevalence of obstructive sleep apnoea syndrome (OSAS) is between 100% of cases as reported by Lin et al. [14] and 33% of MPS patients as described by Kiely et al. [3]. The prevalence of OSAS in patients with MPS was reported as 69.8% by Moreira et al. [39]. Upper airway obstruction has been described for all MPS disorders [40] (Table 2). Other studies in the literature report intermediate percentages [1–3, 12–15, 41–43] (Table 3). MPS I and MPS VI patients appear to be at the greatest risk for severe OSA [1, 15, 42].

**Table 1:** Respiratory disorders in MPS patients: a review of the literature

	No. of patients	MPS type (*n*)	Respiratory disorders
Kiely, 2017 [3]	55	MPS I	RI (54.5%) Snoring (85.5%) OSA (32.7%) Laryngomalacia (9.1%)
Cohen, 2017 [34]	43	MPS III	RI (34.88%) OSA (30.23%) T&A (34.88%)
Chiong, 2017 [35]	23	MPS II	UAO (39%) RI (78.3%) OSA (39%) T&A (39%)
Lin, 2014 [36]	35	MPS I (1) MPS II (12) MPS III (4) MPS IV (16) MPS VI (6)	T&A (96%) OSA (71%) Laryngomalacia (38%)
Muhlebach, 2013 [37]	31	MPS I (9) MPS II (19) MPS III (1) MPS IV (1) MPS VI (1)	T&A (72%) Bronchomalacia (46%) Laryngomalacia (31%)
Wold, 2010 [9]	9	MPS I (5) MPS II (3) MPS VI (1)	UAO (44.4%)
Yeung, 2009 [29]	27	MPS IH (8) MPS IH/S (3) MPS II (5) MPS III B (1) MPS VI (1)	UAO (70%) RI (47%)
Bredenkamp, 1992 [38]	45	MPS IH (13) MPS II (7) MPS III (12) MPS IV (6) MPS VI (5) MPS VII (2)	UAO (38%)
Semenza, 1988 [17]	21	MPS IH (4) MPS IH/S (3) MPS II (3) MPS IV (6) MPS VI (3) MPS VII (4)	UAO (50–90%) T&A (67%) Supraglottic narrowing (92%) OSA (89%)

*MPS* mucopolysaccharidosis, *OSA* obstructive sleep apnoea, *RI* respiratory infection, *T&A* adenoid and/or tonsillar hypertrophy, *UAO* upper airway obstruction,

**Table 2:** Respiratory manifestations in classic/severe forms of MPS disorders

MPS	Upper airway obstruction	Lower airway obstruction	Restrictive lung disease
I	+++	+++	+++
II	+++	+++	++
III	Minimal	Minimal	Minimal
IV	*++*	*+*	*+++*
VI	*+++*	*+++*	*++*
VII	*+++*	*+++*	*++*

Data from Rapoport et al. [40]

*MPS* mucopolysaccharidosis

**Table 3:** Obstructive sleep apnoea (OSA) distribution and severity in each type of MPS

	No. of patients	MPS type (*n*)	OSA prevalence	OSA rating
Kiely, 2017 [3]	55	MPS I	32.7%	NA
Gonuldas, 2014 [1]	76 (only 42 studied with polysomnography)	MPS I (8) MPS II (9) MPS III (23) MPS IV (13) MPS VI (21) MPS VII (2)	95%	Mild (40.47%) Moderate (14.28%) Severe (40.47%)
Morimoto, 2014 [41]	35	MPS I (5) MPS II (25) MPS III (2) MPS IV (2) MPS VI (1)	43%	NA
Kasapkara, 2014 [42]	19	MPS I (4) MPS II (4) MPS VI (11)	94.7%	Mild (26.3%) Moderate (10.5%) Severe (57.9%)
Mesolella, 2013 [2]	20	MPS I (7) MPS II (6) MPS III (4) MPS IV (1) MPS VI (2)	45%	NA
John, 2011 [12]	28	MPS VI	85.1%	Mild (14.28%) Moderate (17.85%) Severe (50%)
Lin, 2010 [14]	24	MPS I (3) MPS II (15) MPS III (1) MPS IV (1) MPS VI (4)	100%	Mild (9.1%) Moderate (31.81%) Severe (59.09%)
Nashed, 2009 [15]	11	MPS IH (4) MPS IH/S (2) MPS II (3) MPS IV (2)	64%	Mild (9.09%) Moderate (27.27%) Severe (27.27%)
Schwartz, 2007 [43]	120	MPS II	48%	NA
Leighton, 2001 [13]	26	MPS I (10) MPS II (6) MPS III (4) MPS IV (4) MPS VI (2)	92%	NA

*MPS* mucopolysaccharidosis, *NA* not available

### What is the possible management for respiratory disorders in MPS?

Currently, treatment of airway obstruction in patients with MPS is controversial, and has met with only moderate success [29]. Management of airway involvement begins with the surgical removal of the obstruction [37], and the accumulation of GAGs in the adenoids and tonsils, with resulting hypertrophy, makes these structures frequent targets for surgical intervention [2]. Therefore, MPS patients with clinical symptoms of upper airway obstruction are often subjected to adenoidectomy and/or tonsillectomy, with data reported in the literature ranging from 70 to 20% of cases [1–3, 7, 12, 19, 29, 34, 37, 42] (Table 4). Tonsil volume increase is also secondary to GAG deposits in the tonsillar fossa. In principle, this would make coblation intracapsular tonsillectomy not recommendable for treatment of OSA in MPS children [38]. Adenoidectomy and/or tonsillectomy in 35–50% of cases are performed prior to MPS diagnosis [7, 44]. Despite adenotonsillectomy being a routine procedure in most children, the risks are usually higher in an MPS child, including post-operative haemorrhage, airway oedema, and failure to extubate [10]. Therapeutically, adenoidectomy and tonsillectomy provide initial but only temporary relief of upper airway obstruction. Furthermore, the recurrence rate of adenoid hypertrophy after adenoidectomy in the MPS population is 56% [1, 45], while in the normal population it is between 0.55 and 1.5% [42]. Adenotonsillectomy alone may not be a sufficient treatment for upper airway obstruction in patients with MPS. The limited relief afforded by adenotonsillectomy is attributable to the multifactorial pathogenesis of airway obstruction and the benefit of the procedure also depends on the extent and severity of airway narrowing at other levels [10].

**Table 4:** MPS and adenotonsillectomy

	No. of patients	MPS type (*n*)	Surgery (%)
Cohen, 2017 [34]	43	MPS III	T&A (23.25%)
Kiely, 2017 [3]	55	MPS I	A (72.7%) T (61.8%)
Gonuldas, 2014 [1]	76	MPS I (8) MPS II (9) MPS III (23) MPS IV (13) MPS VI (21) MPS VII (2)	T&A (34.21%)
Kasapkara, 2014 [42]	19	MPS I (4) MPS II (4) MPS VI (11)	A (26.3%) T (5.3%) T&A(15.8%)
Muhlebach, 2013 [37]	31	MPS I (9) MPS II (19) MPS III (1) MPS IV (1) MPS VI (1)	T&A (16.12%)
Mesolell, 2013 [2]	20	MPS I (7) MPS II (6) MPS III (4) MPS IV (1) MPS VI (2)	A (15%) T (10%) T&A (25%)
Malik, 2013 [19]	10	MPS II	T&A (70%)
John, 2011 [12]	28	MPS VI	A (21.4%%) T&A (10.7%)
Mendelsohn, 2010 [7]	527	MPS II	A (49.5%) T (35.5%)
Yeung, 2009 [29]	27	MPS IH (8) MPS IH/S (3) MPS II (5) MPS IIIB (1) MPS VI (1)	T&A (70.37%)

*A* adenoidectomy, *MPS* mucopolysaccharidosis, *T* tonsillectomy, *T&A* adenotonsillectomy

When local airway procedures are no longer adequate, or when there is significant tracheobronchial involvement, non-invasive continuous positive airway pressure (CPAP) may be employed by some patients during sleep [19] and it is often added to the management plan [37]. It may provide temporary relief for some of these patients before surgical intervention, but it is generally poorly tolerated in patients with behavioural disturbance [10] and may become less effective as the airway disease progresses [29]. If patients tolerate CPAP but hypoxaemia persists, bi-level positive airway pressure (BiPAP) can be advantageous in patients with additional respiratory failure and baseline low oxygen levels.

**Fig. 4:**
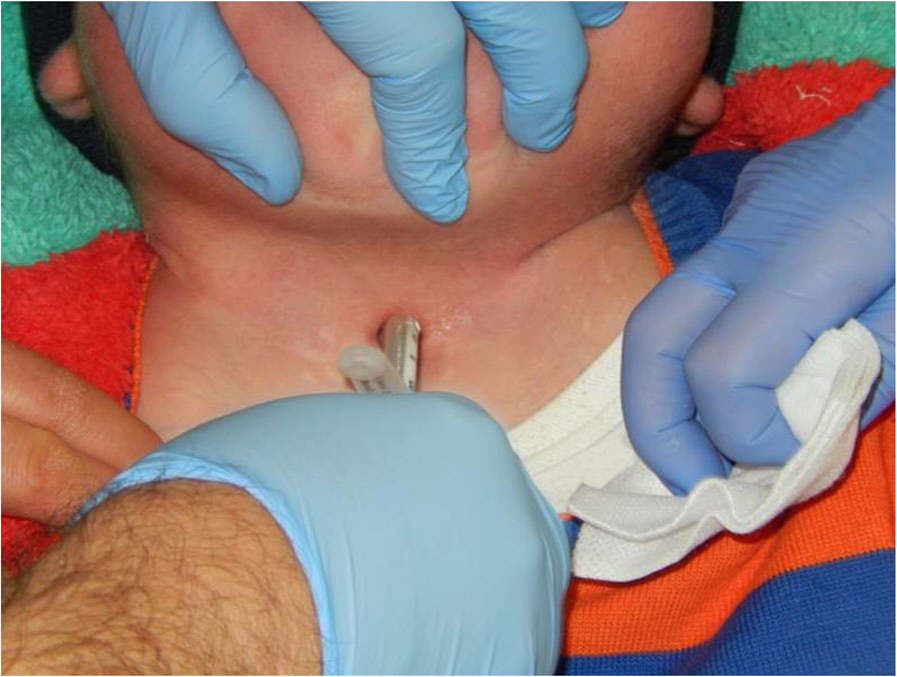
Stomal narrowing

Progression of upper airway obstruction when less invasive interventions are no longer adequate may often require a tracheotomy. Tracheotomy in MPS children may be performed to treat refractory progressive upper airway obstruction, to safeguard an anticipated difficult airway management before a planned non-ENT surgical procedure, and in emergency airway management. Yeung et al. report a rate of 11% for tracheotomy [29]. This rate compares with those in other studies reported in the literature [2, 7, 12, 19, 29, 37, 38, 46] (Table 5). Computed tomographic scan of the chest and airway is recommended prior to surgery to exclude airway narrowing distally from the carina [26] and multidetector computed tomography (MDCT) is suggested for pre-operative airway assessment [47]. MDCT images produce additional information on the glottic and subglottic structures, and airway reconstruction using MDCT derived from previous CT studies could provide a useful assessment tool in the pre-operative airway evaluation and planning of anaesthesia for MPS children. All tracheotomies in patients with MPS result in tracheotomy-related complications. Infrastomal tracheal stenosis is the most frequent complication (85.7%), and stomal narrowing (Fig. 4) also occurs frequently (71.4%) after each tracheotomy [46]. Granulation formation and wound infection are described [18]. These complications cause difficult cannula care, with a high frequency of surgery revisions. Airway problems may persist after tracheotomy due to persistent airway collapse distal to the tip of the endotracheal tube [21], and tracheobronchial stents may be useful [48, 49].

**Table 5:** MPS and tracheotomy

	No. of patients	MPS type (*n*)	Tracheotomy (%)
Muhlebach, 2013 [37]	31	MPS I (9) MPS II (19) MPS III (1) MPS IV (1) MPS VI (1)	19.35%
Mesolella, 2013 [2]	20	MPS I (7) MPS II (6) MPS III (4) MPS IV (1) MPS VI (2)	5%
Malik, 2013 [19]	10	MPS II	100% Complications: Granulation (40%) Wound infection (30%) Ulceration (10%)
John, 2011 [12]	28	MPS VI	3.6%
Mendelsohn, 2010 [7]	527	MPS II	4.4%
Yeung, 2009 [29]	27	MPS IH (8) MPS IH/S (3) MPS II (5) MPS III B (1) MPS VI (1)	11%
Jeong, 2006 [46]	3	MPS II	100% Complications: Stomal narrowing (71.4%) Granulation (57.1%) Wound infection (28.6%) Infrastomal stenosis (85.7%)
Bredenkamp, 1992 [38]	45	MPS IH (13) MPS II (7) MPS III (12) MPS IV (6) MPS VI (5) MPS VII (2)	16%

*MPS* mucopolysaccharidosis

### Is hearing loss an important symptom for the diagnosis of MPS?

**Fig. 5:**
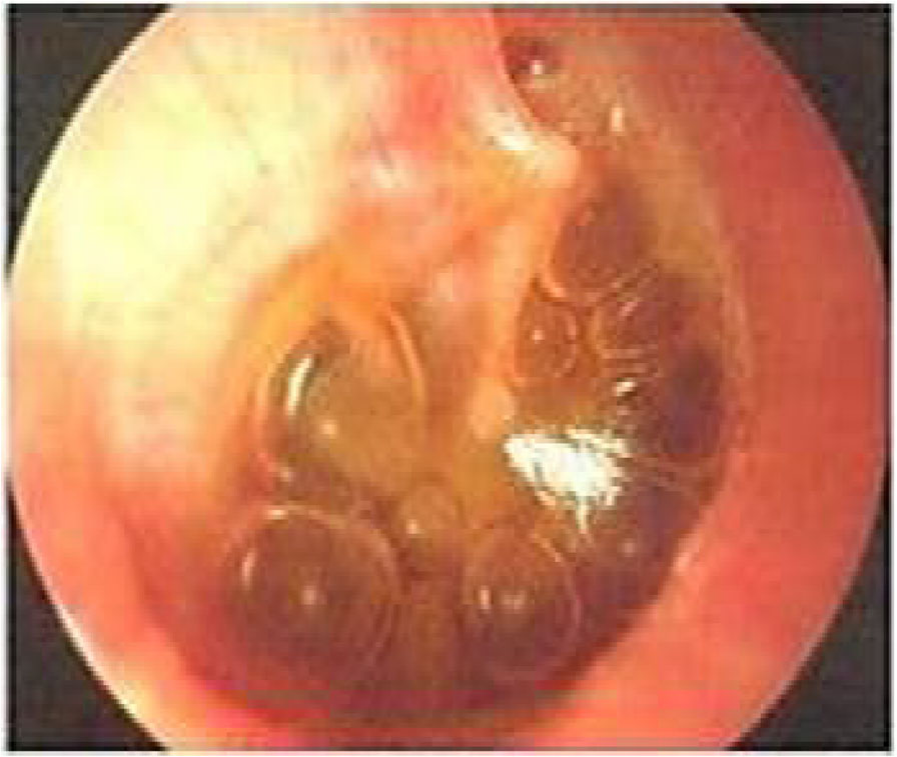
Middle ear effusion

Hearing loss is almost a universal finding in children with MPS [29, 50, 51]. From the hearing standpoint, it is very common to find conductive hearing loss. However, many patients present a sensorineural component [52]. The conductive component of hearing loss is attributed to the presence of seromucinous otitis (Fig. 5) or bone chain deformities [53], disruption in ossicular conduction by histopathological anomalies similar to otosclerosis, or by arthropathy [54]. Sensorineural hearing loss (SHL) is thought to be caused by the accumulation of GAGs in the cochlea, auditory nerve, and brain stem. Auditory pathophysiology in the central nervous system in Hurler syndrome remains unknown [55]; however, decreased cochlear hair cells may be one of the important factors for the sensorineural component of hearing loss. In many patients, mixed-type hearing loss can also be seen. In MPS II (Hunter syndrome), the most prevalent otolaryngological manifestations and interventions reported are otitis (either acute otitis media or chronic otitis media [72%]), hearing loss (67%), insertion of ventilation tubes (50%), adenoidectomy (47%), and hearing aids (41%) [5]. Tables 6 and 7 describe the different audiological findings in subtypes of MPS [52, 56].

**Table 6:** Audiological findings in subtypes of MPS

Sign/symptom	MPS I	MPS II	MPS III	MPS IV	MPS VI	MPS VII
Hearing loss	++	++	+	+	++	++
Recurrent ear infections	++	++	+	+	++	++

*MPS* mucopolysaccharidosis

*++* exhibited by majority of patients with diagnosis, + exhibited by some patients with diagnosis

**Table 7:** Audiometrical findings of patients with MPS

Patient	Age (years)	Diagnosis	Tympanometrical findings		Type of hearing loss		Degree of hearing loss	
			Right	Left	Right	Left	Right	Left
1	9	MPS VI	Type C	Type A	Conductive	Conductive	Slight	Mild
2	4	MPS VI	Type C	Type C	Mixed	Mixed	Moderate	Moderate
3	3	MPS I	Type B	Type B	Mixed	Mixed	Severe	Severe
4	3	MPS I	Type B	Type B	Mixed	Mixed	Severe	Severe
5	9	MPS VI	Type B	Type B	Conductive	Conductive	Moderate	Moderately severe
6	8	MPS IV	Type B	Type B	Mixed	Mixed	Moderate	Mild
7	6	MPS I	Type B	Type B	Conductive	Conductive	Moderate	Moderately severe
8	7	MPS IV	Type B	Type B	Mixed	Mixed	Moderate	Moderate
9	2	MPS III	Type B	Type B	Mixed	Mixed	Severe	Profound

*MPS* mucopolysaccharidosis

### Are these signs typical of early-onset MPS?

Early recognition of MPS requires careful attention to the presence of multiple signs and symptoms, many of which overlap with common childhood complaints. Children with MPS have a high risk of hearing loss and this is an early symptom. Thus, early otolaryngological evaluation and intervention are recommended [56]. Clinical suspicion of the disease can be triggered by particular clusters of signs and symptoms that are unlikely to appear in an unaffected child but that often occur together in the child with MPS II (Table 8).

**Table 8:** “Red flag” signs and symptoms of MPS II that occur early in the disease course

Coarse facial features (may be subtle in the attenuated phenotype)	
Recurrent respiratory infections	
Chronic rhinorrhoea	
Upper airway restriction/noisy breathing/snoring	
Recurrent otitis media	
Hearing loss	
Heart murmur	
Hepatomegaly	
Umbilical and inguinal hernia	
Recurrent watery diarrhoea	
Joint stiffness	
Developmental delay and/or speech delay (in severe phenotype only)	

*MPS* mucopolysaccharidosis

### What is the therapeutic treatment for these audiological-otologic findings?

**Fig. 6:**
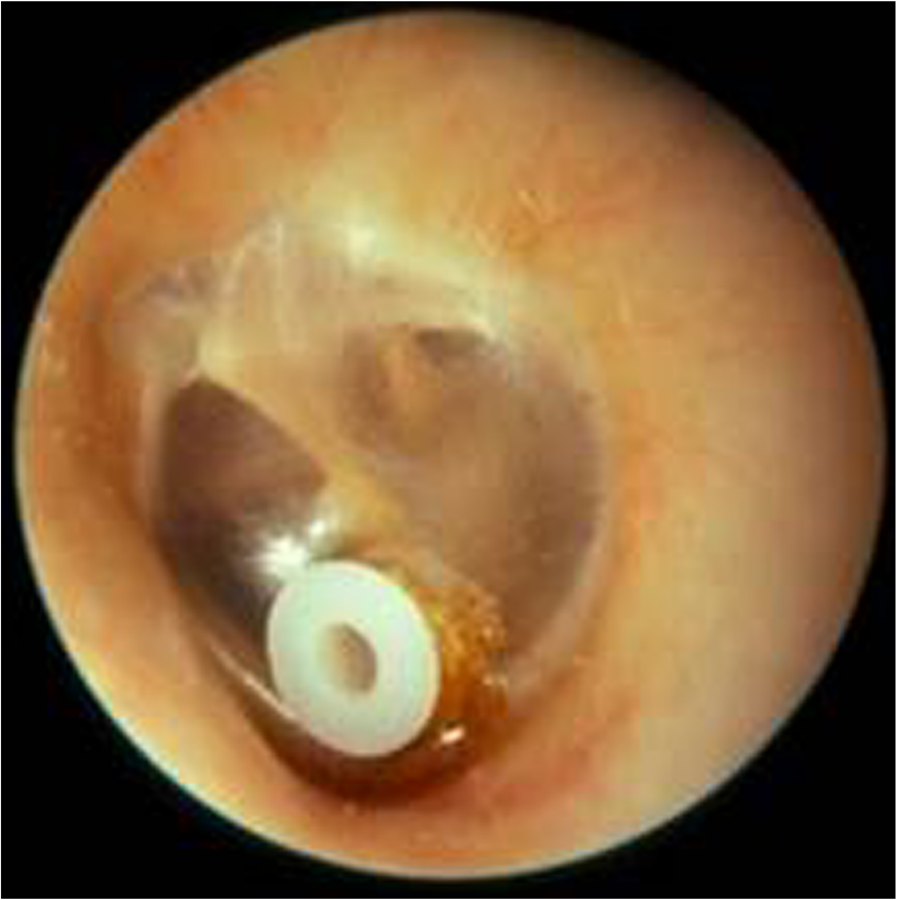
Ventilation tube

Sensorineural or mixed conductive and sensorineural hearing loss commonly develop in Morquio A patients in the first decade of life [57]. Conductive hearing loss due to retained middle ear fluid can be treated using ventilation tubes. Long-lasting types of tympanostomy tubes (Fig. 6) are preferable for use on the first occasion considering the anaesthetic risks and the risk of the re-occurrence of the middle ear fluid. Post-aural hearing aids may be most appropriate if a progressive neurosensory element to hearing loss is present. However, despite hearing normalisation after placement of transtympanic drainage tubes, this does not exempt such patients from a periodic audiological follow-up [51, 58]. Both amplification with hearing aids and transtympanic ventilation tubes appear to be effective in improving language development in children with moderate cognitive impairment [59]. Periodic follow-up of these patients is mandatory because of hearing impairment and consequences for their development and quality of life.

## Conclusion

MPS are rare, progressive, and multisystem diseases with insidious signs and symptoms. Various ENT manifestations appear in the early stage of MPS, including otitis media, macroglossia, adenotonsillar hypertrophy, nasal obstruction, OSAS, progressive respiratory disorders, and hearing loss. As the incidence of hearing loss is high in MPS patients, hearing loss should be determined at an early stage. After the diagnosis, the required treatments should be started promptly with the aim of increasing the quality of life. However, it has to be considered that this is only possible as a result of regular and systematic follow-up. Respiratory disorders occur in all types of MPS. The respiratory involvement is usually progressive and can result in morbidity and mortality early in childhood [10, 26]. MPS patients with clinical symptoms of upper airway obstruction often undergo adenoidectomy and/or tonsillectomy (20–70% of cases). Adenoidectomy and/or tonsillectomy in 35–50% of cases are performed prior to MPS diagnosis [7, 44]. Progression of upper airway obstruction when less invasive interventions are no longer adequate may often require a tracheotomy. All tracheotomies in patients with MPS result in tracheotomy-related complications.

Undiagnosed MPS patients are frequently referred to otolaryngologists because of their early onset of ENT manifestations, before the diagnosis of MPS is confirmed. Otolaryngologists thus have an early opportunity to recognize MPS and they can play an increasingly integral role in the multidisciplinary approach to the diagnosis and management of many children with MPS. The ENT commitment is therefore to suspect MPS when non-specific ENT pathologies are associated with repeated surgical treatments, unexplainable worsening of diseases despite correct treatment, and with signs, symptoms, and pathological conditions such as hepatomegaly, inguinal hernia, macrocephaly, macroglossia, coarse facial features, hydrocephalous, joint stiffness, bone deformities, valvular cardiomyopathy, carpal tunnel syndrome, and posture and visual disorders. Prompt diagnosis may also help avoid complications during surgical intervention. Patients with MPS submitted to surgery may experience difficulty or complications with all kinds of surgical procedures, possibly leading to emergency tracheotomy or even death. If a patient is recognized pre-operatively to have MPS, a well-prepared team of anaesthesiologists and surgeons can be primed for any surgical intervention required and they will know to use specific surgical and intubation/extubation techniques that are recommended for patients with MPS [60].

Therefore, we suggest that otolaryngologists should consider MPS for an early detection of a possible MPS-affected child when collecting the medical history of all children, and especially those being sent to an ENT specialist earlier than usual (at 2–3 years of age) for adenotonsillectomy. The possible association with recurrent otitis media with effusion, inguinal or umbilical hernia, adenotonsillar hypertrophy, sensorineural/conductive hearing loss, OSAS, worsening respiratory obstruction, and bone/joint anomalies should be emphasised. Future studies should consider the use of sleep disordered breathing as an objective parameter of clinical and metabolic improvement [61].

## Abbreviations

CPAP: Continuous positive airway pressure
ENT: Ear, nose, and throat
GAG: Glycosaminoglycan
MDCT: Multidetector computed tomography
MPS: Mucopolysaccharidosi(e)s
OSA: Obstructive sleep apnoea
OSAS: Obstructive sleep apnoea syndrome

## References

[1] Gonuldas B¸, Yılmaz T, Sivri SH, Gucer KS, Kılınc K, Genc GA (2014). Mucopolysaccharidosis: otolaryngologic findings, obstructive sleep apnea and accumulation of glucosaminoglycans in lymphatic tissue of the upper airway. Int J Pediatr Otorhinolaryngol.

[2] Mesolella M, Cimmino M, Cantone E, Marino A, Cozzolino M, Della Casa R (2013). Management of otolaryngological manifestations in mucopolysaccharidoses: our experience. Acta Otorhinolaryngol Ital.

[3] Kiely BT, Kohler JL, Coletti HY, Poe MD, Escolar ML (2017). Early disease progression of hurler syndrome. Orphanet J Rare Dis.

[4] Guffon B, Heron B, Chabrol B, Feillet F, Montauban V, Valayannopoulos V (2015). Diagnosis, quality of life, and treatment of patients with hunter syndrome in the French healthcare system: a retrospective observational study. Orphanet J Rare Dis.

[5] Keilmann A, Iain TN, Bruce A, Molte D, Malm G (2012). Hearing loss in patients with mucopolysaccharidosis II: data from HOS—the hunter outcome survey. J Inherit Metab Dis.

[6] Scarpa M, Almássy Z, Beck M, Bodamer O, Bruce IA, De Meirleir L (2011). Mucopolysaccharidosis type II: European recommendations for the diagnosis and multidisciplinary management of a rare disease. Orphanet J Rare Dis.

[7] Mendelsohn NJ, Harmatz P, Bodamer O, Burton BK, Giugliani R, Jones SA (2010). Importance of surgical history in diagnosing mucopolysaccharidosis type II (hunter syndrome): data from the hunter outcome survey. Genet Med.

[8] Wraith JE, Beck M, Giugliani R, Clarke J, Martin R, Muenzer J, Investigators HOS (2008). Initial report from the hunter outcome survey. Genet Med.

[9] Wold SM, Derkay CS, Darrow DH, Proud V (2010). Role of the pediatric otolaryngologist in diagnosis and management of children with mucopolysaccharidoses. Int J Pediatr Otorhinolaryngol.

[10] Muhlebach MS, Wooten W, Muenzer J (2011). Respiratory manifestations in mucopolysaccharidoses. Paediatr Resp Rev.

[11] Arn P, Bruce A, Wraith JE, Travers H, Fallet S (2015). Airway-related symptoms and surgeries in patients with mucopolysaccharidosis. Ann Otol Rhinol Laryngol.

[12] John A, Fagondes S, Schwartz I, Azevedo AC, Barrios P, Dalcin P (2011). Sleep abnormalities in untreated patients with mucopolysaccharidosis type VI. Am J Med Genet Part A.

[13] Leighton SEJ, Papsin B, Vellodi A, Dinwiddie R, Lane R (2001). Disordered breathing during sleep in patients with mucopolysaccharidoses. Int J Pediatr Otorhinolaryngol.

[14] Lin HY, Chen MR, Lin CC, Chen CP, Lin DS, Chuang CK (2010). Polysomnographic characteristics in patients with mucopolysaccharidoses. Pediatr Pulmonol.

[15] Nashed A, Al-Saleh S, Gibbons J, MacLusky I, MacFarlane J, Riekstins A (2009). Sleep-related breathing in children with mucopolysaccharidosis. J Inherit Metab Dis.

[16] Santamaria F, Andreucci MV, Parenti G, Polverino M, Viggiano D, Montella S (2007). Upper airway obstructive disease in mucopolysaccharidoses: polysomnography, computed tomography and nasal endoscopy findings. J Inherit Metab Dis.

[17] Semenza GL, Pyeritz RE (1988). Respiratory complications of mucopolysaccharide storage disorders. Medicine.

[18] Berger KI, Fagondes FC, Giugliani R, Hardy KA, Sheng Lee K, McArdle C (2013). Respiratory and sleep disorders in mucopolysaccharidosis. J Inherit Metab Dis.

[19] Malik V, Nichani J, Rothera MP, Wraith JE, Jones SA, Walker R, Bruce IA (2013). Tracheostomy in mucopolysaccharidosis type II (Hunter’s syndrome). Int J Pediatr Otorhinolaryngol.

[20] Nagano R, Takizawa S, Hayama N, Umemura S, Uesugi T, Nakagawa S (2007). Three-dimensional CT and histopathological findings of airway malacia in hunter syndrome. Tokai J Exp Clin Med.

[21] Pelley CJ, Kwo J, Hess DR (2007). Tracheomalacia in an adult with respiratory failure and Morquio syndrome. Respir Care.

[22] S.-L. Shih, Y.-J. Lee, S.-P. Lin, C.-Y. Sheu, Blickman J.G. (2002). Airway changes in children with mucopolysaccharidoses: CT evaluation. Acta Radiologica.

[23] Sims HS, Kempiners JJ (2007). Special airway concerns in patients with mucopolysaccharidoses. Respir Med.

[24] Simmons MA, Bruce IA, Penney S, Wraith E, Rothera MP (2005). Otorhinolaryngological manifestations of the mucopolysaccharidoses. Int J Pediatr Otorhinolaryngol.

[25] Shapiro J, Strome M, Crocker AC (1985). Airway obstruction and sleep apnea in hurler and hunter syndromes. Ann Otol Rhinol Laryngol.

[26] Shinhar SY, Zablocki H, Madgy DN (2004). Airway management in mucopolysaccharide storage disorders. Arch Otolaryngol Head Neck Surg.

[27] Parini R, Rigoldi M, Tedesco L, Boffi L, Brambilla A, Bertoletti S (2015). Enzymatic replacement therapy for hunter disease: up to 9 years experience with 17 patients. Mol Genet Metab Rep.

[28] Parini R, Jones SA, Harmatz PR, Giugliani R, Mendelsohn NJ (2016). The natural history of growth in patients with hunter syndrome: data from the hunter outcome survey (HOS). Mol Genet Metab.

[29] Yeung AH, Cowan MJ, Horn B, Rosbe KW (2009). Airway management in children with mucopolysaccharidoses. Arch Otolaryngol Head Neck Surg.

[30] Giugliani R, Harmatz P, Wraith JE (2007). Management guidelines for mucopolysaccharidosis VI. Pediatrics.

[31] Walker R, Belani KG, Braunlin EA, Bruce IA, Hack H, Harmatz PR (2013). Anaesthesia and airway management in mucopolysaccharidosis. J Inherit Metab Dis.

[32] Reichert R, Campos LG, Vairo F, de Souza CF, Pérez JA, Duarte JÁ (2016). Neuroimaging findings in patients with mucopolysaccharidosis: what you really need to know. Radiographics.

[33] Dempsey JA, Veasey SC, Morgan BJ, O’Donnell CP (2010). Pathophysiology of sleep apnea. Physiol Rev.

[34] Cohen MA, Stuart GM (2017). Delivery of anesthesia for children with mucopolysaccharidosis type III (Sanfilippo syndrome): a review of 86 anesthetics. Pediatr Anesth.

[35] Chiong MA, Canson DM, Abacan MAR, Baluyot MMP, Cordero CP, Silao CL (2017). Clinical, biochemical and molecular characteristics of Filipino patients with mucopolysaccharidosis type II—hunter syndrome. Orphanet J Rare Dis.

[36] Lin SP, Shih SC, Chuang CK, Lee KS, Chen MR, Niu DM (2014). Characterization of pulmonary function impairments in patients with mucopolysaccharidoses—changes with age and treatment. Pediatr Pulmonol.

[37] Muhlebach MS, Shaffer CD, Georges L, Abode K, Muenzer J (2013). Bronchoscopy and airway management in patients with mucopolysaccharidoses (MPS). Pediatr Pulmonol.

[38] Bredenkamp JK, Smith ME, Dudley JP, Williams JC, Crumley RL, Crockett DM (1992). Otolaryngologic manifestations of mucopolysaccharidoses. Ann Otol Rhinol Laryngol.

[39] Moreira GA, Kyosen S, Patti C, Martins AM, Tufik S (2014). Prevalence of obstructive sleep apnea in patients with mucopolysaccharidosis types I, II, and VI in a reference center. Sleep Breath.

[40] Rapoport D, Mitchell J (2017). Pathophysiology, evaluation, and management of sleep disorders in the mucopolysaccharidoses. Mol Genet Metab.

[41] Morimoto N, Kitamura M, Kosuga M, Okuyama T (2014). CT and endoscopic evaluation of larynx and trachea in mucopolysaccharidoses. Mol Genet Metab.

[42] Kasapkara CS, Tümer L, Aslan AT, Hasanoğlu A, Ezgü FS, Küçükçongar A (2014). Home sleep study characteristics in patients with mucopolysaccharidosis. Sleep Breath.

[43] Schwartz I, Ribeiro MG, Mota JG, Toralles MB, Correia P, Horovitz D (2007). A clinical study of 77 patients with mucopolysaccharidosis type II. Acta Paediatr.

[44] Keilmann A, Lassig AK, Pollak-Hainz A, Mann WJ, Beck M, Hainz M (2015). Adenoids of patients with mucopolysaccharidoses demonstrate typical alterations. Int J Pediatr Otorhinolaryngol.

[45] Monroy A, Behar P, Brodsky L (2008). Revision adenoidectomy—a retrospective study. Int J Pediatr Otorhinolaryngol.

[46] Jeong HS, Cho DY, Ahn KM, Jin DK (2006). Complications of tracheotomy in patients with mucopolysaccharidoses type II (hunter syndrome). Int J Pediatr Otorhinolaryngol.

[47] Ingelmo PM, Parini R, Grimaldi M, Mauri F, Romagnoli M, Tagliabue G (2011). Multidetector computed tomography (MDCT) for preoperative airway assessment in children with mucopolysaccharidoses. Min Anestesiol.

[48] Rutten M, Ciet P, van den Biggelaar R, Oussoren E, Langendonk JL, van der Ploeg AT, Langeveld M (2016). Severe tracheal and bronchial collapse in adults with type II mucopolysaccharidosis. Orphanet J Rare Dis.

[49] Karl R, Carola S, Regina E, Thomas N, Huber RM (2016). Tracheobronchial stents in mucopolysaccharidosis. Int J Pediatr Otorhinolaryngol.

[50] Santos S, López L, González L, Domíngueza J (2011). Hearing loss and airway problems in children with mucopolysaccharidoses. Acta Otorrinolaringol Esp.

[51] Motamed M, Thorne S, Narula A (2000). Treatment of otitis media with effusion in children with mucopolysaccharidoses. Int J Pediatr Otorhinolaryngol.

[52] Gökdoğan C, Altinyay S, Gökdogan TH, Gündüz B, Okur I (2016). Audiologic evaluations of children with mucopolysaccharidosis. Braz J Otorhinolaryngol.

[53] Zechner G, Moser M (1987). Otosclerosis and mucopolysaccharidosis. Acta Otolaryngol.

[54] Netzloff ML, Elsea SH, Fisher RA. Genetic hearing loss associated with metabolic disorders. In: Toriello, Reardon, Gorlin (eds). Hereditary hearing loss and its syndromes. 2nd ed. Oxford University Press; 2004:387–392.

[55] Kariya S (2012). Inner ear changes in mucopolysaccharidosis type I/Hurler syndrome. Otol Neurotol.

[56] Burton BK, Giuliani R (2012). Diagnosing hunter syndrome in pediatric practice: practical considerations and common pitfalls. Eur J Pediatr.

[57] Hendriksz CJ, Berger KI, Giugliani R, Harmatz P, Kampmann C, Mackenzie WG (2015). International guidelines for the management and treatment of Morquio a syndrome. Am J Med Genet.

[58] Cho YS, Kim JH, Kim TW, Chung SC, Chang SA, Jin DK (2008). Otologic manifestations of hunter syndrome and their relationship with speech development. Audiol Neuro Otol.

[59] Vargas-Gamarra MF (2017). Audiological findings in children with mucopolysaccharidoses type I–IV. Acta Otorrinolaringol Esp.

[60] Walker RW, Darowski M, Morris P, Wraith JE (1994). Anaesthesia and mucopolysaccharidoses. A review of airway problems in children. Anaesthesia.

[61] Pal A, Langereis E, Saif M, Mercer J, Church H, Tylee K (2015). Sleep disordered breathing in mucopolysaccharidosis I: a multivariate analysis of patient, therapeutic and metabolic correlators modifying long term clinical outcome. Orphanet J Rare Dis.

